# Vertically Aligned CdO-Decked α-Fe_2_O_3_ Nanorod Arrays by a Radio Frequency Sputtering Method for Enhanced Photocatalytic Applications

**DOI:** 10.1021/acsomega.2c02996

**Published:** 2022-08-03

**Authors:** Mansour Alhabradi, Srijita Nundy, Aritra Ghosh, Asif Ali Tahir

**Affiliations:** † Environment and Sustainability Institute, University of Exeter, Penryn TR10 9FE, United Kingdom; ‡ Department of Physics, Faculty of Science, Majmaah University, Majmaah 11952, Saudi Arabia; § College of Engineering, Mathematics and Physical Sciences, Renewable Energy, University of Exeter, Cornwall TR10 9FE, U.K.

## Abstract

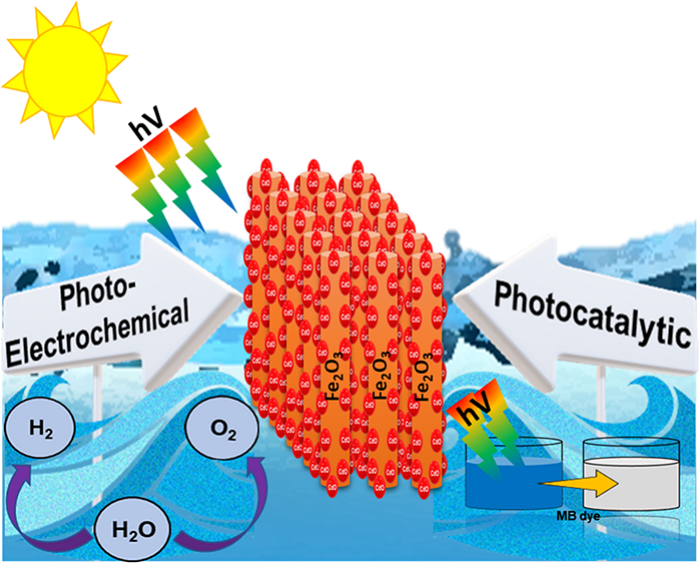

Green hydrogen production is one of the most desirable sustainable goals of the United Nations. Thus, for that purpose, we developed hematite (α-Fe_2_O_3_), an n-type semiconductor, a desirable candidate for photoelectrochemical (PEC) water splitting, enabling hydrogen evolution. High recombination losses, low efficiency, and large-scale production hinder its potential. To address these issues, we have fabricated optimized bare and cadmium oxide (CdO)-decorated hematite thin film nanorod arrays using a throughput radio frequency (RF) sputtering with efficient water splitting behavior. To the best of our knowledge, no work has been done so far on the synthesis of CdO/α-Fe_2_O_3_ via RF sputtering for PEC application. Bare α-Fe_2_O_3_ samples, with a morphology of vertically aligned nanorods, were fabricated with optimized parameters such as as-deposited 70 nm of Fe, an angle of deposition of 70°, and an annealing temperature of 600 °C, which showed a photocurrent density of 0.38 mA/cm^2^ at 1.65 V vs reversible hydrogen electrode (RHE). Characterizations depicted that this unique morphology with high crystallinity directly enhanced the performance of hematite photoanodes. Further, deposition of 30 nm of cadmium (CdO) on the α-Fe_2_O_3_ nanorods produced a corn-like morphology with CdO nanoparticles (∼2 nm), resulting in 4-times enhancement of the PEC performance (1.2 mA/cm^2^ at 1.65 V vs RHE). CdO acted as a co-catalyst, responsible for satisfactory suppression of recombination and facilitating the hole transfer, directly enhancing the overall photocurrent density. This photoanode showed an extremely stable behavior over a period of 26 h when kept under constant illumination. Furthermore, the CdO-modified photoanode showed a better dye degradation (98% in 40 min) than the bare hematite (60% in 40 min), proving to be an efficient photoanode.

## 1 Introduction

Green hydrogen (H_2_), the most sustainable and cleaner form of energy, is regarded an emergent future fuel capable of replacing traditional fossil fuels and achieving a zero carbon emission goal.^1^ This is in accordance with the United Nation’s 17 sustainable development goals, which are targeted at the development of a better future with affordable and clean energy (goal 7). By 2030, EU, UK, and China aim toward producing 6GW, 10GW, and 100GW power from hydrogen production, respectively.^2^ “Hydrogen economy” is a global interminable solution with increasing climate change and energy demands.^3^ Hence, development of green and sustainable H_2_ fuel is an important ongoing research, which is still facing challenges such as controlled production, transportation, and safe storage.^4^

Solar-assisted water splitting, popularly known as photoelectrochemical (PEC) water splitting, is the most promising, cost effective, and environmentally friendly approach for generation of hydrogen. Since 1972, Fujishima and Honda^5^ and many other researchers investigated a variety of materials such as TiO_2_,^6^ ZnO,^7^ Cu_2_O,^8^ WO_3_,^9^ Fe_2_O_3_,^10^ and BiVO_4_^11^ for PEC application, where α-Fe_2_O_3_ (hematite) gained interest due to its suitable bandgap of 2.1 eV,^12,13^ capable of absorbing a substantial fraction of solar irradiation (40% energy from visible light). Hematite is a very stable n-type semiconductor popular not only in the field of photocatalysis but have also been extensively used in the field of sensors,^14^ batteries,^15^ and wastewater treatment^16^ due to its visible light-absorbing ability. However, the main drawback of hematite is its large charge recombination and slower surface kinetics, which diminishes the resultant photocurrent.^17^ Furthermore, researchers also established that the PEC performance of hematite anodes can be greatly improved with development of nanostructures such as nanorods, nanotubes, nanowires, and nanoparticles.^18−20^ Development of these nanostructures will enable the reduction of scattering of the carriers due to their higher surface area, which is advantageous for the PEC efficiency.^21^ Several nanofabrication methods have been employed such as atmospheric pressure-assisted chemical vapor deposition,^22^ spray pyrolysis,^23^ solgel,^24^ reactive magnetron sputtering,^25^ aerosol-assisted CVD,^26^ ferrocene,^1^ hydrothermal,^27^ etc. for the development of hematite-based photoanodes, which will enable the tuning of the surface morphology and composition, which would directly affect and enhance their PEC performance. Although most of the processes are time-consuming and result in production of non-uniform coating, physical vapor deposition using radio frequency (RF) magnetron sputtering is a throughput approach, which seemed to be the most versatile, uniform, large-scale, and rapid deposition technique also suitable for nanostructure development with parameter modulation.^28^ Among many nanostructures, 1D nanostructured morphologies such as nanorods, nanobelts, nanofibers, and nanowires have gained a lot of interest in this field due to their sizes, which if comparable to the length of hole diffusion, can efficiently reduce the recombination problem and also enhance the PEC performance of the hematite. Thus, growing vertically aligned nanorods efficiently is of research interest nowadays. Most of the researchers employ a hydrothermal method for the development of vertically aligned hematite nanorods on any kind of conducting surface, and few works have reported the growth of vertically aligned hematite using an RF PVD method. A glancing angle deposition technique (GLAD) is one of the approaches opted by some researchers to grow WO_3_ nanorods on FTO via the PVD sputtering method.^28,29^ However, no work has been reported for hematite growth using this technique. Apart from morphology variation, surface modification with doping, decoration, or even formation of heterostructures is another potential approach to improve the PEC efficiency of hematite photoanodes.^30,31^ Researchers synthesized a variety of heterostructure photocatalysts such as Fe_2_O_3_/TiO_2_,^30^ Fe_2_O_3_@SnO_2_,^32^ Fe_2_O_3_/CdS,^33^ and ZrO_2_-Fe_2_O_3_^34^ by impregnating growth of TiO_2_, SnO_2_ CdS, and ZrO_2_ onto Fe_2_O_3_ or incorporating Zr,^35^ Ag,^36^ Ti,^37^ and Pt^38^ on hematite to form doped hematite. Results showed a significant improvement in their photocatalytic performance as compared to pristine Fe_2_O_3_ by improving the charge separation between electrons and holes. Among these materials, cadmium sulfide (CdS) has been extensively coupled as a heterostructure to hematite due to its wide bandgap (2.42 eV) and similar conduction and valence band positions, resulting in a fast charge separation at the interface. However, CdS is photocorrosive in aqueous solution. Thus, an alternative to it can be cadmium oxide (CdO), an n-type semiconductor with a direct bandgap of 2.5 eV at room temperature.^39^ CdO is predominantly used as a transparent conducting oxide electrode, photovoltaic, photodiode, phototransistor, IR detector, and anti-reflection coating^40^ and is individually an excellent photocatalyst. Thus, CdO can be coupled with Fe_2_O_3_ to enhance the overall efficiency.

Herein, we employed RF magnetron sputtering for the development of high-quality vertically aligned hematite nanorod films for photocatalysis application from pure iron (Fe) sputter followed by high-temperature annealing, which is a more cost-effective process than the other approaches mentioned above. Most reports used Fe_2_O_3_ targets along with oxygen supply for the development of such thin films, making it a very expensive approach. Nanostructured hematite thin films of varied thickness were developed by augmenting the processing parameters such as annealing temperature and angle of deposition. Finally, the PEC performance of the as-developed photoanodes was carried out. The effect of varied deposition parameters on the development of hematite thin films with a rod-like morphology and their corresponding PEC performance was identified, analyzed, and discussed in detail. Further, with the best optimized hematite thin film, cadmium was deposited on it, which oxidized in air at room temperature to give cadmium oxide on the hematite thin films. The obtained samples were characterized by X-ray diffraction (XRD), scanning electron microscopy (SEM) coupled with energy-dispersive X-ray spectroscopy (EDX), transmission electron microscopy (TEM), and UV–vis absorption spectroscopy. The enhanced PEC efficiency was notable, and the detailed mechanism was discussed, supported with characterization and experimental validation.

## 2 Experimental Section

### 2.1 Synthesis of RF Sputtered High-Quality Fe_2_O_3_ Thin Films

**Figure 1:**
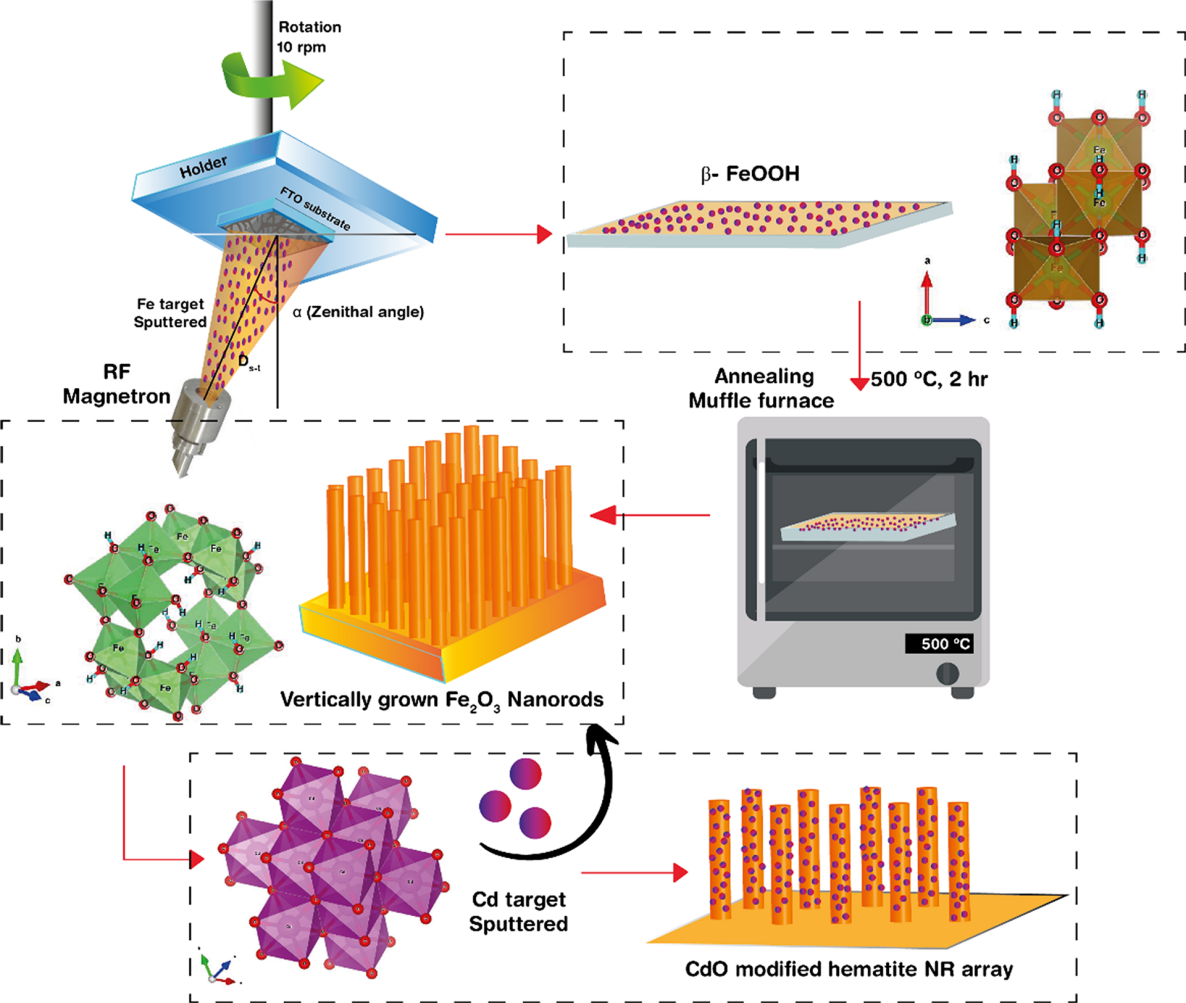
Fabrication of vertically aligned RF sputtered high-quality Fe_2_O_3_ nanorod thin films.

High-quality nanorod hematite thin film photoanodes for PEC analysis were developed by depositing a pure iron (Fe) target (3″ × 1/16″ sputtering target – Fe, 99.95% purity, Moorfield Nanotechnology) on a 1 by 2 cm fluorine-doped tin oxide (FTO, NSG TEC 5, Pilkington)-coated glass substrate at room temperature by means of RF magnetron sputtering (Figure 1). Prior to use, all FTO substrates were thoroughly cleaned by means of ultrasonication with deionized water (DIW), ethanol, and acetone followed by drying. The distance between the FTO substrate and the Fe target (*d*_s-t_) was maintained at 15 cm, and the zenithal angle of deposition (α) was varied from 25 to 80° during deposition to obtain a nanostructured morphology. The sputtering was executed under vacuum with a working pressure of 0.3 Pa after introducing 20 sccm of 99.99% pure Ar gas into the deposition chamber. The rate of deposition and the thickness were monitored using a quartz crystal microbalance. Prior to the Fe deposition on the substrate, pre-sputtering was conducted for 5 min, with the shutter protection between the target and the substrate, as an enhanced cleaning process. During the Fe deposition process, the substrate rotation was kept constant at 10° and the RF sputtering power was varied from 30 to 70 W. To obtain the best PEC performance, different samples were prepared with varied Fe film thickness from 10 to 150 nm. The as-obtained samples were taken for high-temperature annealing in a muffle furnace at 500–650 °C for 2 h at a rate of 5°/min to obtain vertically aligned hematite nanorods. The samples were allowed to cool down naturally to room temperature before being taken for any characterization or experiments. The best optimized hematite thin film was then again exposed to Cd deposition using pure 3″ × 1/16″ Cd sputtering target (99.95% purity, Moorfield Nanotechnology) with various thicknesses (10, 15, 20, 25, 30, 35, 40, 45, and 50 nm) at a rate of deposition of 1.2 Å/s. The as-obtained samples were taken for high-temperature annealing in a muffle furnace at 450 °C for 1 h at a rate of 5°/min to obtain CdO-decorated hematite films, and after cooling down to room temperature, they were taken for characterization and PEC measurements.

### 2.2 Thin Film Characterization

The crystal structures and the phases of the as-synthesized hematite thin films were characterized using a Bruker D8 X-ray diffractometer assisted with monochromatic Cu-Kα (λ = 0.154 nm) radiation. Scanning electron microscopy (FE-SEM) using a TESCAN VEGA3 was used to study the surface morphology of the films along with energy-dispersive spectroscopy (EDS) with Oxford Instruments to study and analyze the elements present in the synthesized films. Detailed microstructural analysis was conducted using low- and high-resolution transmission electron microscopy (HR-TEM) with a JEOL JEM-2100F with 200 kV. The optical bandgap and transmission spectra were obtained from a UV–VIS–NIR UV-3600 Plus spectrophotometer.

### 2.3 Photoelectrochemical (PEC) Measurements

Finally, all photoelectrochemical studies of the hematite thin films were conducted utilizing a Metrohm Autolab (PGSTAT302N) workstation using a three-electrode compartment (platinum = counter electrode, Ag/AgCl in 3 M KCL = reference electrode, FTO-coated hematite thin film = working electrode, 1 M NaOH with a pH of 13 = electrolyte). The intensity of light was simulated to achieve the 1 sun condition (100 mW/cm^2^) using a Newport 66902, 300 W xenon lamp with an AM 1.5 filter and 420 nm cutoff filter to remove the UV part of sunlight. The potentials of the hematite photoanodes (potential vs Ag/AgCl) were recorded at a scan rate of 0.01 V/s from the cathodic to anodic potential direction (−0.3 V and +0.7) under chopping conditions. All potentials were converted to the reversible hydrogen electrode (RHE) potential according to Nernst equation (eq 2)
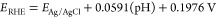
1

Further, the Mott–Schottky relationship was employed to determine the flat band potential (*V*_fb_) of both pure and CdO-decorated hematite nanostructured films using the equation below (eq 3):
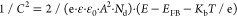
2where *C* is the space charge capacitance, ε (80 for Fe_2_O_3_) and ε_0_ are the dielectric constant of the semiconductor and the permittivity of free space (8.854 × 10^–12^ F/m), e is the electron charge, *A* is the area of the thin film, *N*_d_ is the dopant density, *E* and *E*_FB_ are the applied and flat band potential, and *T* and *K*_b_ are the temperature and Boltzmann constant (1.38 × 10^–23^ J/K), respectively.

### 2.4 Photocatalytic Dye Degradation Test

The photocatalytic behavior of as-synthesized bare and CdO-modified hematite thin films against methylene blue (MB) dye degradation under visible irradiation was studied with a 300 W ozone free xenon lamp (with AM 1.5 filter, Newport 66902) at room temperature. The lamp was adjusted to obtain the 1 sun condition (100 mW/cm^2^). Pure MB stock solution was prepared by dissolving 10 mg of MB powder in 1 L of DIW to obtain 10 mg/L concentration. The as-obtained thin films were dipped in 30 mL of MB solution and kept in a cylindrical Pyrex under constant stirring and illumination. To attain a stable adsorption/desorption equilibrium, the entire setup was kept in the dark for 30 min prior to illumination. Aliquots of the solution after a regular interval of time were withdrawn and taken for adsorption tests in the UV–Vis spectrophotometer.

The rate of degradation was calculated according to following equation (eq 4):
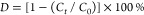
3where *D* is the degradation efficiency and *C*_0_ and *C*_t_ are the initial and final concentrations at time *t* = 0 and *t* = *t* respectively.

## 3 Results and Discussion

### 3.1 Optimizing Parameters for Deposition: Effects of Pressure and Power on the Rate of Deposition

Direct growth of hematite films on FTO-coated low iron glass via an RF magnetron sputtering-assisted physical vapor deposition method involves controlling and optimizing various process parameters, and therefore, analyzing the effect of these modulating factors on the tuneable properties of the as-synthesized films is crucial. The Fe target was sputtered onto the substrate by optimizing the chamber pressure, the rate of deposition, and the RF power. The effect of these parameters directly influenced the deposition time and material properties. First, keeping the *d*_*s*-t_ (150 mm), Ar gas inflow rate (20sccm), angle of deposition (70°), and RF power (30 W) fixed, the chamber pressure was decreased gradually from 3 to 0.3 Pa. Figure S1a demonstrates that with a decrease in the chamber pressure, the rate of deposition linearly increases from 0.12 Å/s (at 3 Pa) to 0.33 Å/s (at 0.3 Pa). Figure S1 also depicts the variation in the rate of deposition as a function of argon flow. Argon gas was varied from 22 to 2 sccm, and it was validated that with an increase in Ar flow, the rate of deposition also increases. Thus, 20 sccm was found to be the best Argon rate, at which the rate of deposition was maintained at 0.33 Å/s. After achieving the optimized chamber pressure (0.3 Pa) and the rate of Ar flow (20 sccm), the effect of RF power on the rate of deposition was also investigated. Figure S1b displays the trend obtained for the rate of deposition of indium tin oxide, niobium, and Fe sputtering targets. With an increase in the RF power from 25 to 75 W (150 W being the maximum RF power, 50% of max RF power should be used to prevent any damage), the rate of deposition constantly increased from 0.3 to 1.2 Å/s. The yield of Fe is 1.2 Å/s at 75 W RF power. Thus, the optimized parameters were established to obtain uniform deposition of hematite thin films of varied thickness ranging from 10 to 150 nm.

### 3.2 Structural and Morphological Analysis

**Figure 2:**
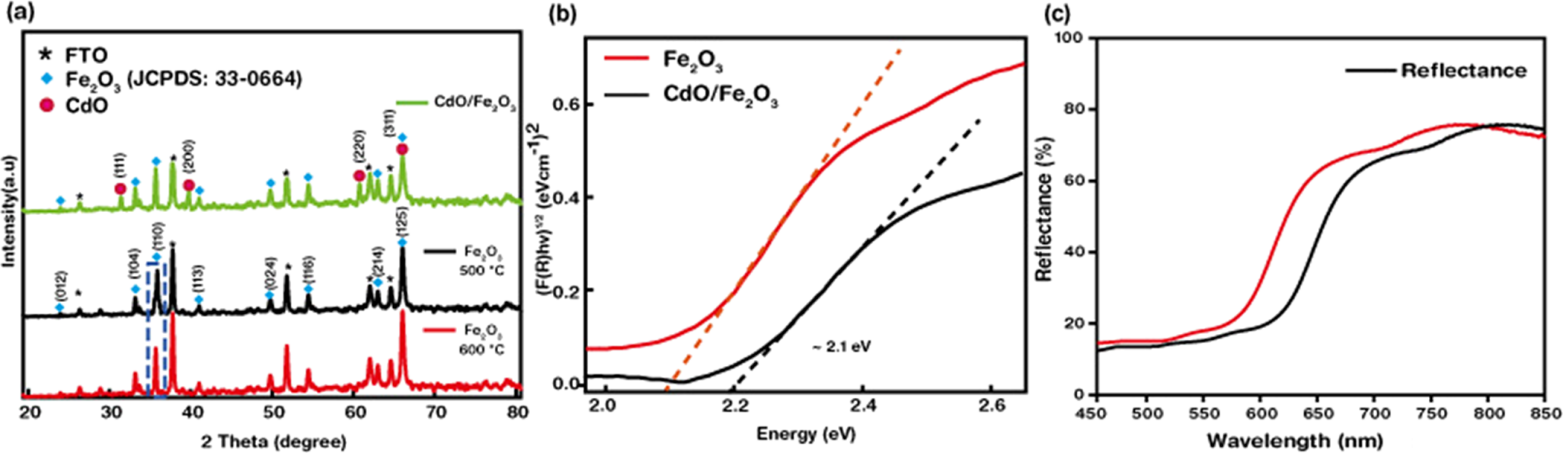
(a) XRD patterns of α-Fe_2_O_3_ nanorods annealed at 600 (red) and 500 (black) and CdO-modified hematite (green) nanorods on the FTO-coated surface. (b) Bandgap estimation of bare ND CdO-decorated α-Fe_2_O_3_ nanorods. (c) Transmission spectra of bare α-Fe_2_O_3_ and CdO-decorated hematite-coated FTO glasses.

Figure 2a displays the XRD patterns of bare (annealed at 500 and 600 °C) and CdO-modified hematite thin films. The analyzed results clearly depict the formation of α-Fe_2_O_3_ (black and red curves) on the FTO substrate due to the presence of sharp and distinct peaks corresponding to the (012), (104), (110), (113), (024), (116), (214), and (214) corresponding to α-Fe_2_O_3_ according to the JCPDS data (33-0664). The peak corresponding to (110) has a strong intensity, signifying the growth along the (110) plane to be dominant. This is because the conductivity of hematite is reported to be the highest along this direction. Also, it can be noted that the XRD pattern points out the narrowing of peaks with an increase in annealing temperature. The hematite film obtained after annealing at 500 °C (black curve) shows a comparatively broad (110) peak, indicating a higher crystallinity than the film annealed at 600 °C (red curve). Also, with an increase in annealing temperature, the lattice strain shrinks or decreases, giving rise to an increase in crystallinity and also resulting in an increase in crystallite size. Next, the XRD results of the CdO-modified hematite thin film (green curve) portray various additional peaks to hematite, which are indexed to CdO (05-0640), corresponding to the (111), (200), (220), and (311) planes, verifying the formation of the CdO/α-Fe_2_O_3_ composite. Additionally, no other peaks were observed, which clearly indicates the absence of other possible impurity phases. Figure 2b displays the direct bandgaps of bare and CdO-modified hematite thin films obtained from the Tauc plot (tangential line drawn to the *x*-axis) to be 2.1 and 2.2 eV, respectively. The bandgap of the modified hematite increased due to addition of CdO, which has an individual bandgap of 2.5 eV. The transmission data shown in Figure 2c shows that the transmission decreases with development of hematite thin films on the FTO-coated glass substrate. The bare FTO glass possessed a transmission (*T*%) of 92%, which decreased to 68% for α-Fe_2_O_3_. This further slightly decreased to 60% for CdO-modified hematite thin films due to formation of CdO nanoparticles and increased surface roughness.

**Figure 3:**
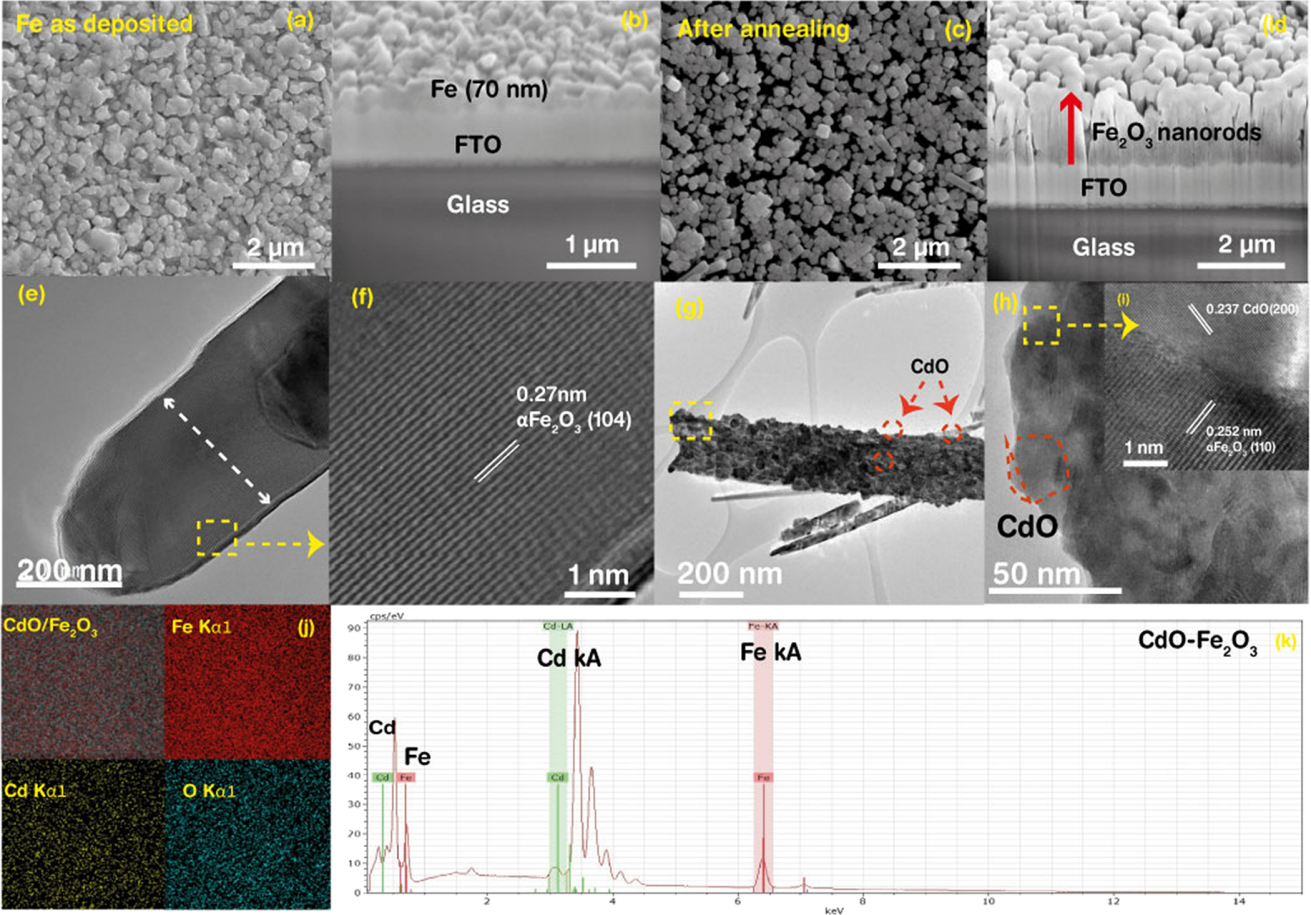
Top and cross-sectional FIB SEM images of (a, b) as-deposited Fe and (c, d) α-Fe_2_O_3_ on the FTO substrate, respectively. Individual TEM and the corresponding HR-TEM images of (e, f) α-Fe_2_O_3_ and (g, h, inset) CdO/α-Fe_2_O_3_ nanorods, respectively. (j) EDS mapping and (k) spectra of the thin film showing the presence of Cd, O, and Fe in CdO-decorated α-Fe_2_O_3_.

The top and cross-sectional views of the SEM images of the as-deposited Fe thin films and α-Fe_2_O_3_ nanorod arrays on the FTO substrate after annealing at 600 °C are shown in Figure 3a–d. Figure 3a,b displays the top and FIB cross-sectional SEM images of the as-deposited Fe thin film, which appears to be agglomerated particle-like with a film thickness of ∼70 nm. After annealing 70 nm of as-deposited Fe thin films at 600 °C, the particles grow into vertically aligned nanorods, uniformly and densely distributed, as shown in Figure 3c,d. From the cross-sectional view (Figure 3d), the formation of uniformly formed vertically aligned nanorods with flat top edges is identified, with a length of 1.2 μm and diameter of 200 nm. Several reports have shown that the formation of these vertically aligned nanorods is due to the optimized angle of deposition^28,41^ (in our case, α = 70°), which resulted in the formation of such morphology. This morphology also contributed to the enhanced PEC efficiency. Further the detailed morphology and microstructure of the bare and CdO nanoparticles (NPs)-decorated hematite nanorods were investigated using low-and high-resolution TEM images and are displayed in Figure 3e–i. Figure 3e demonstrates that the nanorod acquired a smooth surface, and the diameter of each rod was estimated to be ∼200 nm, which were highly crystalline in nature. The HR-TEM images exhibited clear lattice fringes, with a lattice spacing of 0.27 nm, consistent with the α-Fe_2_O_3_ (104) plane, as shown in Figure 3f. Further, low- and HR-TEM images of CdO NPs-decorated hematite nanorods are displayed in Figure 3g–i. We observed the formation of CdO NPs (∼20 nm diameter)-coated hematite nanorod surfaces (Figure 3g), resulting in a very rough surface and corn-like morphology. The HR-TEM image (Figure 3h inset) displays clear lattice fringes with lattice spacing values of 0.237 and 0.252 nm, ascribed to the CdO (200) and α-Fe_2_O_3_ (110) planes, respectively. Finally, the EDS mapping (Figure 3j) and spectra (Figure 3k) of CdO-modified hematite nanorods shown indicates the presence of Cd, Fe, and O in the CdO/α-Fe_2_O_3_ nanocomposite.

### 3.3 Photocatalytic Performance: Effects of Thickness, Annealing Temperature, and Angle of Deposition

**Figure 4:**
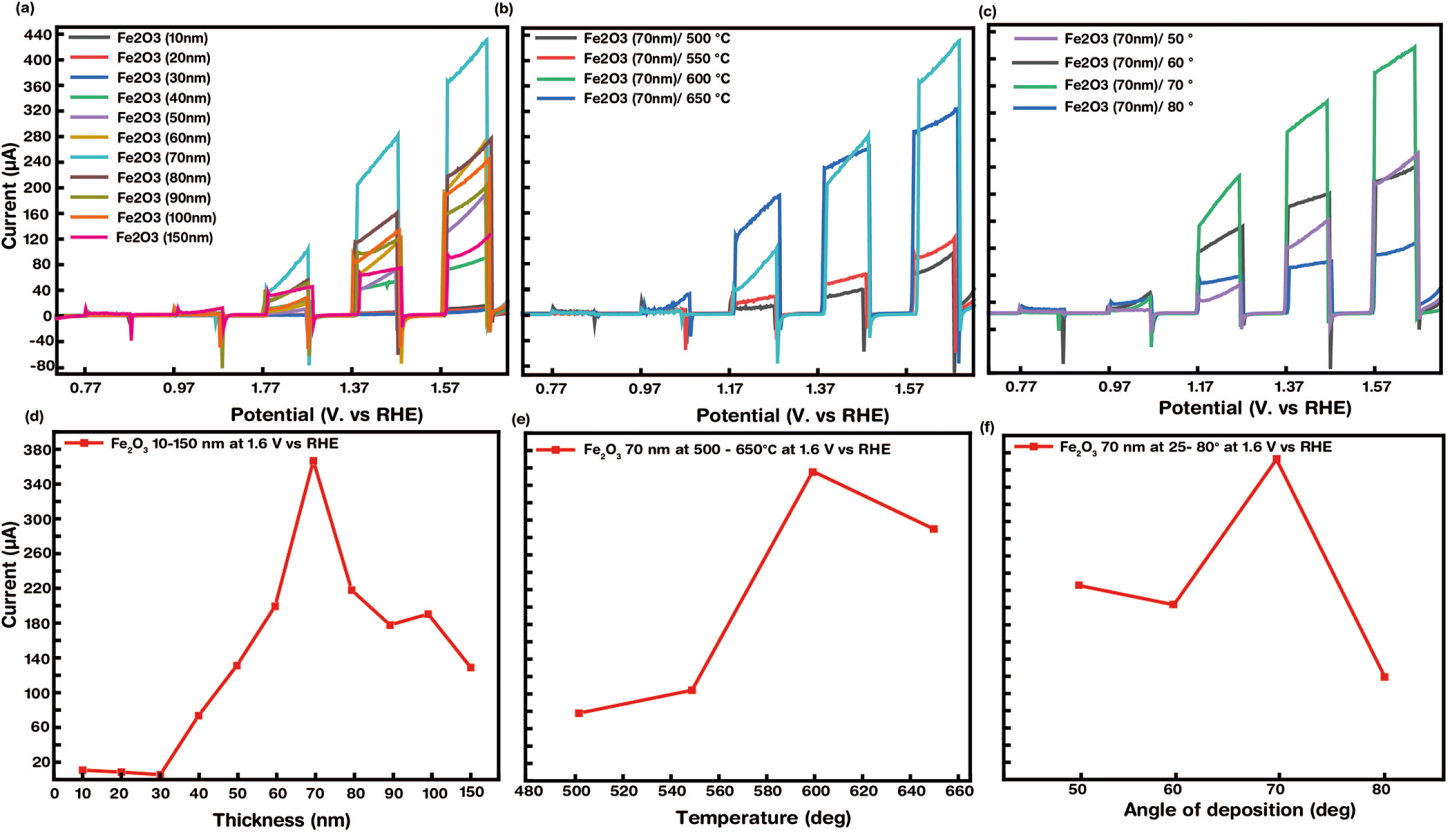
Linear sweep voltammetry (LSV) of current density–potential vs RHE plots under 100 mW/cm^2^ chopped light and dark illumination at a scan rate of 0.1 mV/s in 1 M NaOH electrolyte (pH =13) showing the effect of (a) the as-deposited Fe thickness from 10 to 150 nm, (b) annealing temperature from 500 to 650 °C, and (c) angle of deposition on the PEC performance of hematite nanorod thin film arrays and (d–f) the corresponding observed trends at 1.6 V vs RHE, respectively.

To explore the impact of film thickness on the PEC performance, a large number of hematite thin films were fabricated and annealed at 600 °C with as-deposited Fe thicknesses ranging from 10 to 150 nm, and their corresponding photocurrents were measured. Figure 4a reveals the linear sweep voltage (LSV) plots, i.e., photocurrent (μA) vs potential (vs RHE), at a scan rate of 1 mV s^–1^ of all the as-synthesized α-Fe_2_O_3_ on FTO substrates under chopped dark and solar light illumination conditions. The results clearly indicate that the photocurrent increases for all samples with an increase in the bias voltage from 0.77 to 1.57 V vs RHE. The onset potential was observed from 0.77 V vs RHE, which agrees with the surface kinetics of water oxidation for most hematite films.^30,31^Figure 4b displays the obtained trend of the photocurrent measurements of the samples at their specific thickness. The photocurrent monotonically increased from 20 to 380 μA with an increase in the as-deposited thickness of the Fe film between 10 to 70 nm, respectively. This increment is attributed to the absorption of light by the as-synthesized material. With a further increase in Fe thickness, the photocurrent appears to be reduced significantly. This phenomenon arises due to involvement of grain boundaries and recombination centers. With an increase in Fe deposition beyond 70 nm, agglomeration increases, preventing the growth of nanorods in the suitable direction. Also, the bulk recombination centers become predominant compared to those present in the surface, resulting in an increase in recombination losses, which directly reduces the photocurrent. Thus, the maximum photocurrent of 0.38 mA was obtained for Fe (70 nm), giving rise to vertically aligned hematite nanorods and being the optimized thickness for further measurements. Further, the long-term stability of the α-Fe_2_O_3_ photoanode after 4 months was tested to see no considerable difference in the measurement.

Next, to understand the sensitivity of PEC measurements toward annealing temperature, the α-Fe_2_O_3_ photoanode with 70 nm of as-deposited Fe thickness was annealed at four different temperatures, i.e., 500, 550, 600, and 650 °C. Figure 4c,d shows the typical photocurrent transients, portraying a significant enhancement in the photoactivity (0.08 to 0.38 mA) with the increasing annealing temperature from 500 to 600 °C; this is ascribed to the improved crystallinity of the thin films along with the decrease in defect concentration at the bulk, surface, and interface of FTO and α-Fe_2_O_3_. The presence of defects in hematite results in an increase in the rate of recombination due to a large number of trapped states, thereby directly reducing the photocurrent.^42^ Additionally, a high annealing temperature also contributes toward diffusion of Sn from FTO toward α-Fe_2_O_3_, thereby enhancing the conductivity of the sample. However, a further increase in annealing temperature to 650 °C results in the decrease in photocurrent to 300 μA, which is due to the thermal degradation of the FTO substrate, making them behave as insulators.^43^ The crystallinity of samples at different annealing temperatures can be interpreted from the XRD data (Figure 2a), showing broader peaks for 500 °C and narrower peaks for samples annealed at 600 °C, indicating the improved crystallinity with a higher annealing temperature. Furthermore, the cathodic spikes observed when light is switched off are resultants of hole accumulation at the interface of the electrolyte and α-Fe_2_O_3_, where the rate of photogenerated holes reaching the photoanode/electrolyte interface is more than the rate at which the holes participated in electrochemical reactions at the surface. Thus, a faster photoactivity and high photocurrent (0.38 mA at 1.57 V vs RHE) of the α-Fe_2_O_3_ photoanode with 70 nm of deposited Fe annealed at 600 °C are due to the minimized defect concentration, passivation of interfacial trapped states, larger grain size, and improved crystallinity, directly enhancing the PEC performance.

Finally, the relationship between the angle of deposition (α) and the PEC performance was evaluated by analyzing the photocurrent measurements of the as-prepared samples with varying α values of 25, 50, 60, 70, and 80°. The change in the zenithal or deposition angle influenced not only the photocurrent but also the morphology and growth of α-Fe_2_O_3_. Figure 4e,f represents the photocurrent transients and the trend as a function of angle of sputtering. With an increase in angle from 25 to 70°, the photocurrent gradually increases from 0.22 to 0.38 mA, respectively. This is ascribed to the variation in the growth of the nanorods. The thin films appeared to be smooth and dense with a deposition angle at 25°; further, at 50°, the film showed randomly oriented nanorods; and further, from 60° onward, the surface appeared to be rough and the nanorods were vertically aligned to the surface. Thus, the angle of deposition clearly exhibited morphological alteration, which directly affected the PEC performance. The maximum photocurrent was obtained for the sample deposited at α = 70°, where the nanorods appeared to be vertically oriented. With an increase in the length of nanorods and vertical orientation, the surface area increases and the concentration of photoexcited e–h pairs increases, giving us a superior photocurrent. However, with a further increase in α = 80°, the photocurrent dropped drastically, imputed to an increase in recombination of the charge carriers. Thus, the optimized length of the nanorods obtained at α = 70° provides us with the highest photocurrent.

**Figure 5:**
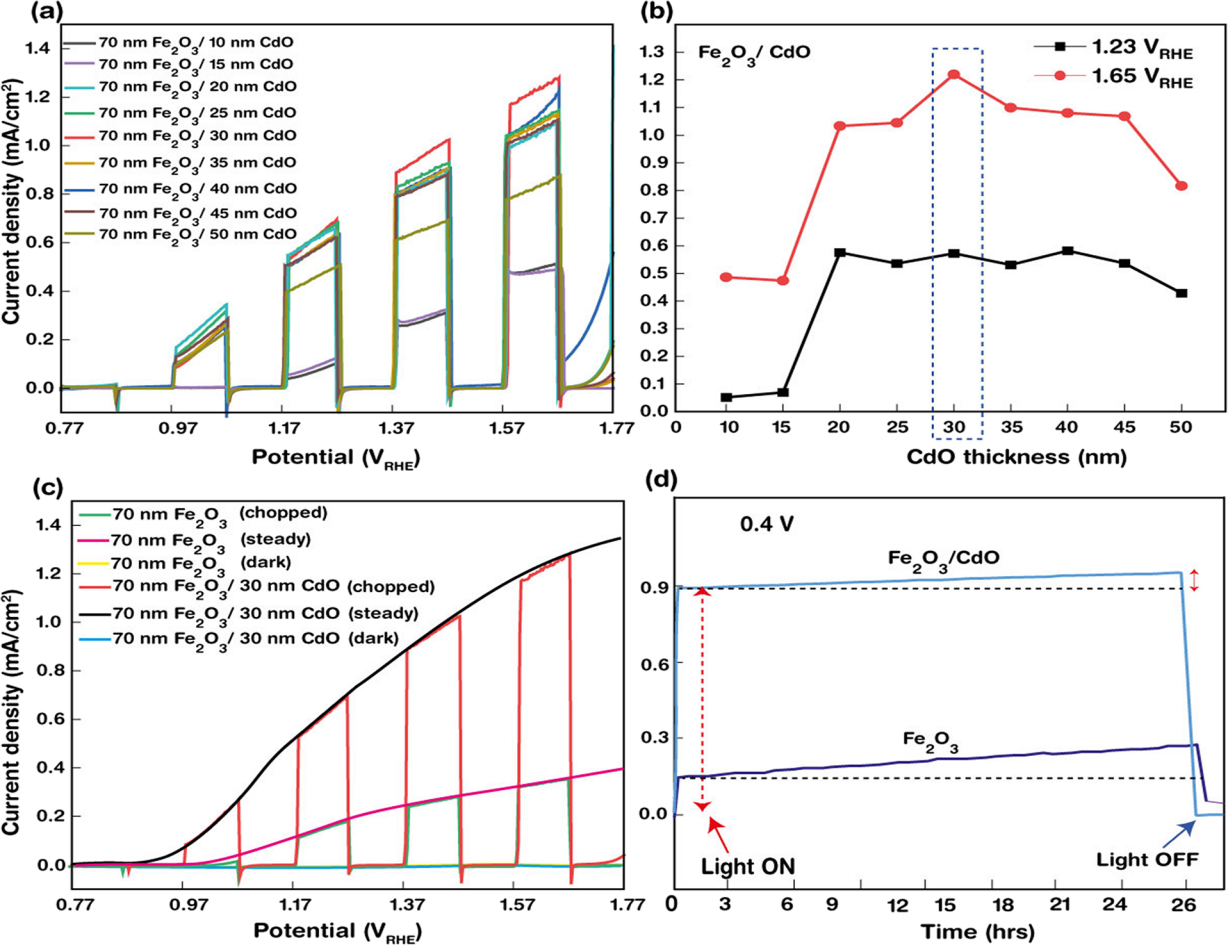
(a) Linear sweep voltammetry (LSV) of current density–potential vs RHE plot under 100 mW/cm^2^ chopped light and dark illumination at a scan rate of 0.1 mV/s in 1 M NaOH electrolyte (pH =13) of CdO-modified hematite thin films with various CdO thicknesses (10 to 55 nm). (b) PEC performance of CdO-modified hematite thin films with different CdO thicknesses at two voltages (1.23 V vs RHE and 1.65 V vs RHE). (c) Comparison of the PEC performance of bare hematite and CdO-modified hematite nanorod thin film arrays. (d) Long-term stability of the fabricated device kept under illumination for 26 h.

Finally, after obtaining the best optimized hematite nanorod array thin film, it was taken for further modification, where Cd was deposited on the films to enhance the overall photocurrent efficiency of the film. The role of CdO (as a co-catalyst) as a function of thickness in the PEC performance was evaluated, and the best optimized results were obtained accordingly. Figure 5a displays the linear sweep voltammetry (LSV) plots of CdO-modified hematite thin films with various as-deposited Cd thicknesses (10 to 50 nm), showing the current density vs potential (vs RHE) data obtained under 100 mW/cm^2^ chopped light and dark illumination at a scan rate of 0.1 mV/s in 1 M NaOH electrolyte (pH = 13). The thickness was noted by adjusting the deposition time during the PVD sputtering process (rate of deposition being 1.2 Å/s). From Figure 5a, it is evident that the photocurrent density of bare hematite films drastically improves with the modification of CdO NPs and thus the PEC performance of Fe_2_O_3_/CdO increases with an increase in the thickness of Cd, with the thickness of Fe_2_O_3_ being constant. The photocurrent density of the composite photoanode increases with an increase in Cd thickness until an optimum thickness of 30 nm, where a maximum photocurrent density of 1.2 mA/cm^2^ (at 1.65 V_RHE_) was acquired, and with a further increase in the thickness beyond 30 nm, the photocurrent decreases to 1.1 mA/cm^2^ (at 1.65 V_RHE_) and then even down to ∼0.55 mA/cm^2^ (at 1.65 V_RHE_) for 35 and 55 nm of Cd, respectively (see Figure 5b), which is still comparatively higher than the bare hematite photoanode. Figure 5c shows a very clear enhancement in the photocurrent density of CdO NPs-decorated Fe_2_O_3_ (1.2 mA/cm^2^ at 1.65 V_RHE_) than the bare Fe_2_O_3_ nanorod photoanode (0.38 mA mA/cm^2^ at 1.65 V_RHE_), which is quite a noteworthy enhancement of ∼4 times. The decrease in PEC performance with thick Cd deposition onto the hematite nanorods originated from weak hole transfer properties of CdO, the valence band position being very near to that of the hematite (see the Mechanism Section). Additionally, a pure CdO-based photoanode showed an extremely poor performance (as shown in Figure S2). Finally, the long-term stability of the photoanode was investigated by keeping it under illumination for 26 h (Figure 5d). A slight insignificant increase in the PEC efficiency is observed over a period of 26 h. Table 1 depicts the synthesis method, PEC performance, and maximum photocurrent achieved by other hematite-based photoanodes as in the contemporary literature.

**Table 1:** Comparison Data Showing the PEC Current Density of Hematite-Based Photoanodes

photoanode	synthesis method	electrolyte	maximum photocurrent density	reference
α-Fe_2_O_3_/CdS	hydrothermal + dip coating	1 M NaOH	0.6 mA/cm^2^ (at 0.92 V vs RHE)	(44)
Si-doped/α-Fe_2_O_3_	electrodeposition + microwave annealing	1 M NaOH	0.45 mA/cm^2^ (at 0.92 V vs RHE)	(45)
Ti-doped/α-Fe_2_O_3_	thermal evaporation	1 M NaOH	0.585 mA/cm^2^ (at 0.92 V vs RHE)	(46)
WO_3_/α-Fe_2_O_3_	sputter deposition	0.5 M NaOH	0.84 mA/cm^2^ (at 0.92 V vs RHE)	(47)
Pt-doped/α-Fe_2_O_3_	magnetron sputtering process and subsequently thermal oxidation	1 M NaOH	0.34 mA/cm^2^ (at 1.23 V vs RHE)	(48)
CdO/α-Fe_2_O_3_	RF magnetron sputtering process and subsequently thermal oxidation	1 M NaOH	1.2 mA/cm^2^ (at 1.65 V vs RHE)	present work

**Figure 6:**
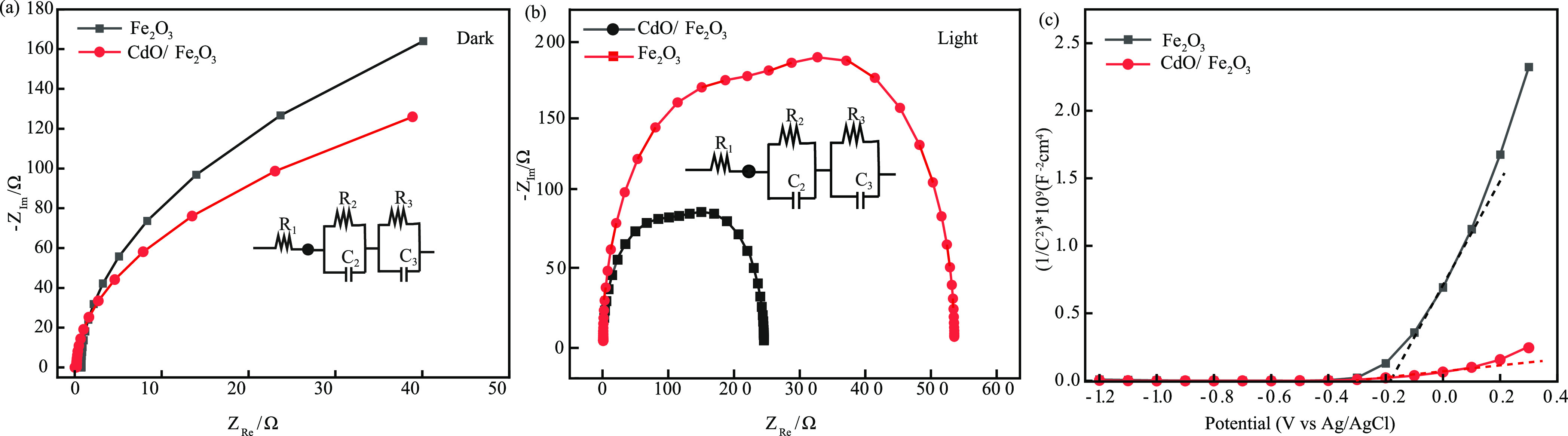
Electrochemical impedance spectroscopy data obtained for pure hematite and CdO-modified hematite photoanodes under (a) dark and (b) light conditions (circuit diagram shown in the insets) along with the (c) Mott–Schottky data showing the flat band potentials.

Further, electrochemical impedance spectroscopy was conducted to investigate the interfacial charge transport kinetics and properties of bare and CdO-modified hematite, which are the key factors responsible for the enhancement of the PEC efficiency. Figure 6a,b shows the Nyquist plots of the electrochemical impedance spectra of bare and CdO-modified hematite photoanode recorded at dark and under solar illumination (100 mW/cm^2^), respectively. The equivalent or the general circuit (*R*_1_ + *R*_2_/*C*_2_ + *R*_3_/*C*_3_) used to interpret the data is shown in the inset of Figure 6a,b, where *R*_1_ indicates the series resistance amidst FTO, connecting wires and hematite, *R*_2_ is the resistance resultant from charge trapping within the accumulation layer between CdO and hematite, *R*_3_ demonstrates the resistance between the catalytic surfaces and the electrolyte, and *C*_2_ and *C*_3_ correspond to the capacitance values of the bulk material and the associated surface states, respectively. After fitting the obtained data with the aforementioned circuit, the values of the fitted parameters according to the circuit are explained below. A semicircle was obtained for both the bare and the CdO-modified photoanodes under illumination, which represents the recombination of photogenerated charge carriers within the material of the photoanodes. It is quite apparent that the CdO-modified hematite photoanode possesses a smaller radius in comparison to Fe_2_O_3_, and the data depicted the charge transfer resistance of bare and CdO-modified photoanodes. The value of *R*_2_ for CdO-decorated hematite is 112.4 Ω, which is comparatively much lower than the bare hematite (235.4 Ω), implying that the formation of CdO NPs on the hematite nanorods results in the reduction of the transfer charge resistance and heightened the charge transport kinetics at the interface caused by the photoanode and the electrolyte. This overall improves the PEC performance of our photoanode by inhibiting the electron–hole pair recombination. Furthermore, it is also observed that the interfacial charge resistance of the modified photoanode (*R*_3_ = 130.2 Ω) decreased compared to the bare hematite (*R*_3_ = 294.7 Ω), which is attributed to the improved ionic conductivity due to decoration with CdO NPs.^44,49,50^

For the Mott–Schottky analysis, the value of the flat band potential is estimated by extrapolating 1/*C*^2^ = 0 (as shown in Figure 6c). The flat band potential of bare hematite is estimated to be −0.2 eV, and that of the CdO modified hematite is −0.4 eV. This shift of the valence flat band indicates that the bending of edges at the electrolyte and electrode interface decreased, which facilitated a better charge transfer. Next, the donor density is calculated from the slope of the Mott–Schottky (1/*C*^2^ vs *V*). The slopes for the bare and CdO NPs-modified hematite nanorods were estimated to be 5.44 × 10^9^ and 6.24 × 10^8^. The lowest slope of the CdO-modified hematite from the plot undoubtedly indicates that decoration with CdO NPs enhanced the density of electron charge carriers, which is known to be inversely proportional to the slope of the plot and the prime reason for the enhanced PEC efficiency.^7^ Thus, the *V*_FB_ and *N*_D_ values of bare and CdO-decorated hematite were obtained as 0.77 V (vs RHE) and 3.24 × 10^20^ cm^–3^ and 0.56 V (vs RHE) and 2.8 × 10^21^ cm^–3^, respectively.^51,52^

**Figure 7:**
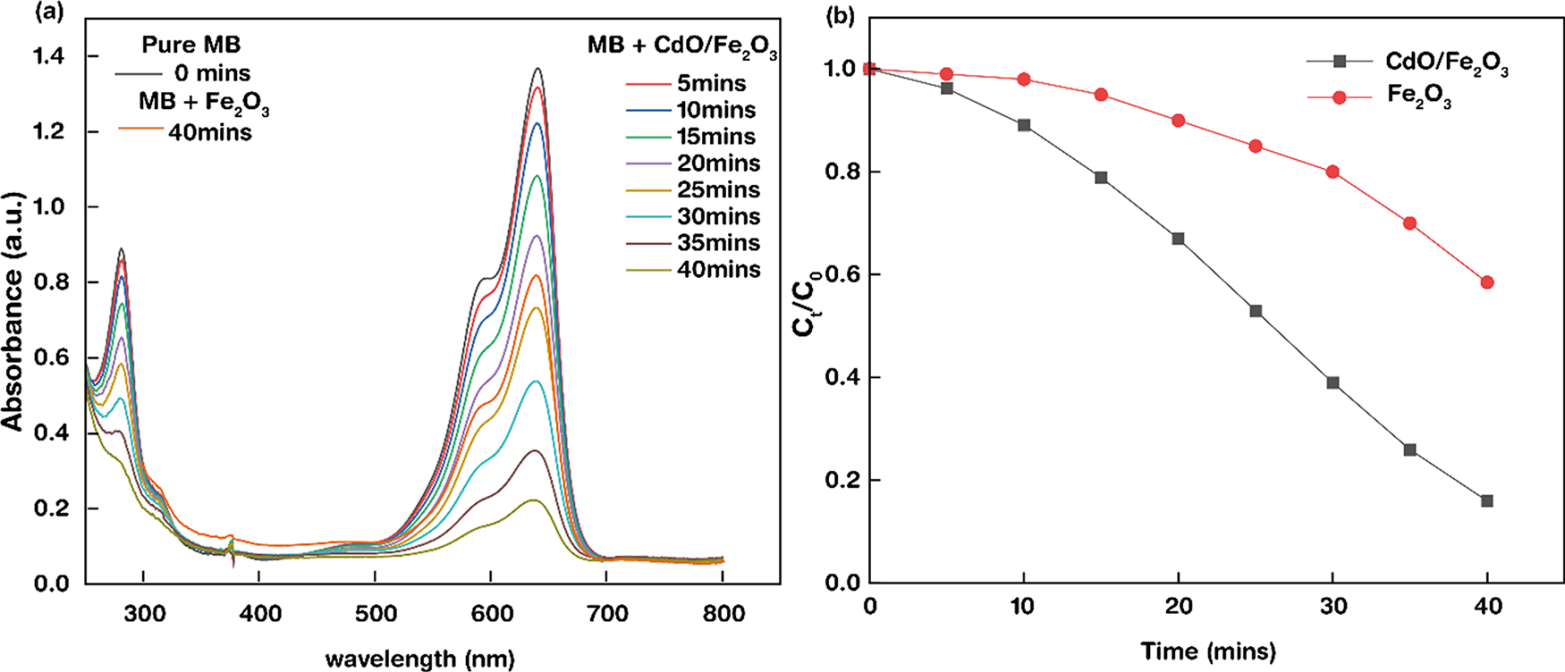
(a, b) Absorbance spectra of methylene blue dye as a function of time as a result of addition of photoanodes (bare and CdO-modified hematite).

Further, the photocatalytic performance of our as-synthesized bare and CdO-modified hematite for the MB dye degradation was studied and evaluated under visible light illumination. Methylene blue being a noxious synthetic dye requires removal from the environment. The absorption intensity of the pure MB dye at 0 min recorded at a wavelength λ of 642 nm gradually decreased, and the degraded absorption intensity of the dye after addition of hematite and CdO-modified hematite was recorded at an interval of 5 min (0–40 min), and the corresponding absorption spectra are displayed in Figure 7a. Figure 7b portrays the *C*_t_/*C*_0_ relation with time, which is required for the efficiency calculation. The degradation efficiency was noted to be 41.5% under the addition of bare hematite, which increased drastically to 84% with the CdO-modified hematite photoanode. Thus, it can be visualized that addition of CdO enhanced the photocatalytic behavior of the hematite, resulting in a better degradation of the MB dye in 40 min. This is attributed to the large specific area providing more active sites for the reaction to occur and effective charge carrier recombination. Additionally, the appropriate conduction and valence band position contribute to the enhancement of the photocatalytic activity.

### 3.4 Photocatalytic Dye Degradation Results

### 3.5 Mechanism

**Figure 8:**
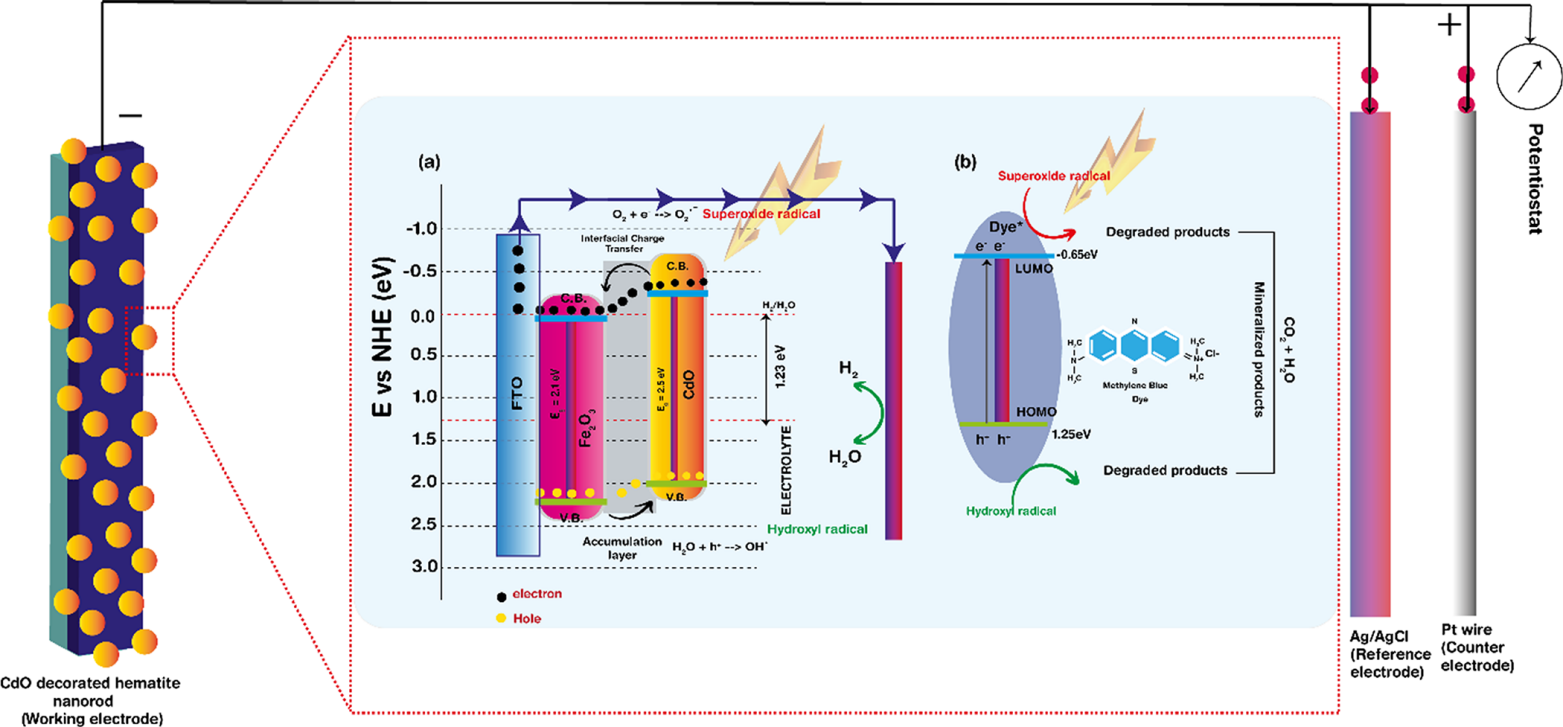
Scheme representation of the CdO-modified α-Fe_2_O_3_ photoanode showing the mechanism involved in (a) photogenerated charge carrier kinetics for photoelectrochemical water splitting and (b) dye degradation applications.

Figure 8a shows the mechanism behind the incredible enhancement in the PEC efficiency, which is mostly attributed to the CdO modification on the hematite via the PVD sputtering method. Various factors are associated with this improvement, such as a shift observed in the onset potential,^53,54^ alteration in band bending at the heterostructure junction,^55^ a decrease in charge recombination,56–^57^ and surface passivation.^56^ In brief, under illumination, electron–hole pairs are generated in the bare hematite photoanode, where photogenerated electrons are transported toward the FTO, thereby contributing toward H_2_O reduction at the cathode surface, alternating producing hydrogen. During this process, a substantial amount of the photocurrent density undergoes recombination losses. Next, the photogenerated holes suffer from slow transfer kinetics at the anode/electrolyte interface, resulting in hole accumulation near the semiconductor surface, resulting in an overall increase in the recombination rate between photogenerated holes and electrons. Thus, bare hematite encounters a substantially slow surface kinetics, resulting in a weak PEC performance. Thus, the surface modification of CdO, which acts as a co-catalyst, is employed to overcome the recombination losses and the slow charge transfer. The highly excited and reactive Cd^2+^ species readily traps the holes from the valence band of adjacent hematite and facilitates the electron transfer, thereby accelerating the H_2_O splitting and enhancing also the number of charge carriers, thereby increasing the current. Thus, CdO modification enables suppressing the recombination and facilitating the hole transfer from the hematite to the electrolyte, hence enhancing the overall PEC efficiency. Thus, with an increase in the thickness of CdO, the active species (Cd^2+^) requires a longer time to oxidize and participate in the H_2_ evolution, attributed to longer decay times, and can result from the formation of intermediate trapped states, which can thermodynamically hinder the hole-transfer kinetics. The calculated valence and conduction band position after modification with CdO directly makes this photoanode suitable as a water splitting photoanode. The reduction in resistances as observed from the impedance data also clearly point to the enhancement of the charge carriers. Thus, an optimized growth of CdO NPs onto the hematite nanorod array thin film results in satisfactory suppression of recombination and direct enhancement in the photocurrent density.

Next, Figure 8b describes the mechanism associated with the photocatalytic activity of the CdO-modified hematite photocatalyst toward MB dye degradation. Under visible solar irradiation, CdO/α-Fe_2_O_3_ results in the formation of photogenerated electron–hole pairs (eq 9), which participates in the reaction accordingly. The electron captures oxygen from the water to produce a superoxide radical (eq 1), whereas the holes are trapped by the surface hydroxyl, resulting in the production of hydroxyl radicals (eq 5). These holes further react with the water molecule to also form hydroxyl radicals (eq 6). These hydroxyl and superoxide radicals contribute toward effective MB dye degradation (eq 8).^58^
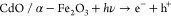
4
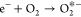
5
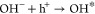
6
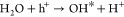
7
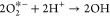
8
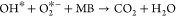
9

## 4 Conclusions

This study depicts the improvement in the photoelectrochemical efficiency of bare hematite after the modification with cadmium oxide NPs. Hematite (α-Fe_2_O_3_) is an n-type semiconductor, with an appropriate wide bandgap of 2.1 eV, which is beneficial for water splitting (1.23 eV), and is able to absorb 40% of solar visible light, directly contributing efficiently toward the PEC water splitting and generation of hydrogen. For large throughput and uniform fabrication of the photoanode, a physical vapor deposition method using RF sputtering was opted to develop high-quality hematite thin film photoanodes. It was observed that deposition parameters played a crucial role in tuning the morphology, crystallinity, and in turn the PEC performance. Hematite thin films were fabricated with varied thickness of Fe ranging from 10 to 150 nm, and it was observed that 70 nm of Fe is the best, at which a high photocurrent density is obtained. Beyond the optimized value, a thicker film showed deterioration due to the formation of grain boundaries and an increase in the recombination centers in the bulk and the surface of the semiconductor. Next, the angle of deposition of the target onto the substrate also played a noteworthy role in the development of the vertically aligned nanorod arrays. The angle of deposition was varied from 25 to 80°, and the photoanode obtained with 70 ° showed very well defined vertically grown nanorod arrays, which also efficiently showed the best photochemical behavior. Finally, the PEC performance sensitivity toward high-temperature annealing was also studied, and results showed that annealing with 600 °C showed a better PEC performance of 0.38 mA/cm^2^ at 1.65 V vs RHE due to increased crystallinity. However, the photocurrent obtained was still low due to recombination losses and weak photogenerated charge transfers. Thus, after optimization of the best hematite photoanode, various thicknesses (10–50 nm) of cadmium were deposited via RF magnetron sputtering on the hematite nanorods, which after annealing, provided CdO NPs on the hematite photoanodes. CdO acted as a co-catalyst in the improvement of the PEC efficiency toward water splitting. CdO deposited on the hematite surface, enriched with highly excited and reactive Cd^2+^ species, facilitated the electron transfer and reduced the interfacial electron–hole pair recombination, thereby enhancing the PEC performance. This unique CdO NPs-decorated vertically aligned hematite nanorods on the FTO substrate, contributed to almost 4-times enhancement in the PEC performance (1.2 mA/cm2 at 1.65 V vs RHE). Additionally, this photoanode proved to be a highly stable photoanode for a period of 26 h under illumination. Furthermore, this photoanode also behaved as an efficient dye degradation photocatalyst toward methylene blue. Addition of CdO (98% in 40 min) enhanced the photocatalytic behavior of the hematite (60% in 40 mins), resulting in a better degradation of the MB dye in 40 min. Thus, our work proposes a scalable approach to fabricate high-quality RF sputtered CdO-modified hematite thin film nanorod arrays for PEC water splitting and dye degradation.

## Supporting Information Available

Variation trends of the rate of deposition of the Fe target as a function of chamber pressure, argon gas inflow, and RF power and LSV plot of Cdo thin films with different thicknesses (current density–potential vs Ag/AgCl) under 100 mW/cm^2^ (PDF)
