# Supplementary material for: Vertically Aligned CdO-Decked α-Fe2O3 Nanorod Arrays by a Radio Frequency Sputtering Method for Enhanced Photocatalytic Applications
Source: ACS Omega. 2022 Aug 3;7(32):28396–407. doi: 10.1021/acsomega.2c02996 (PMC9386802; doi:10.1021/acsomega.2c02996)
Supplement: Supplementary file 1 — ao2c02996_si_001.pdf [file ao2c02996_si_001.pdf]

Supporting Information Only

Vertically aligned CdO decked α-Fe_2O_3 nanorod array by RF sputtering method for enhanced photocatalytic applications

Mansour Alhabradi[1,2], Srijita Nundy[1], Aritra Ghosh[3], Asif Ali Tahir[1,*]

[1]Environment and Sustainability Institute, University of Exeter, Penryn TR10 9FE, United Kingdom

[2]Department of Physics, Faculty of Science, Majmaah University, Majmaah, 11952, Saudi Arabia

[3]College of Engineering, Mathematics and Physical Sciences, Renewable Energy, University of Exeter, Cornwall TR10 9FE, U.K

*Corresponding author: A.A.Tahir (a.tahir@exeter.ac.uk)

There is a total of 2 (two) pages, 2 (two) figures in the supporting information.

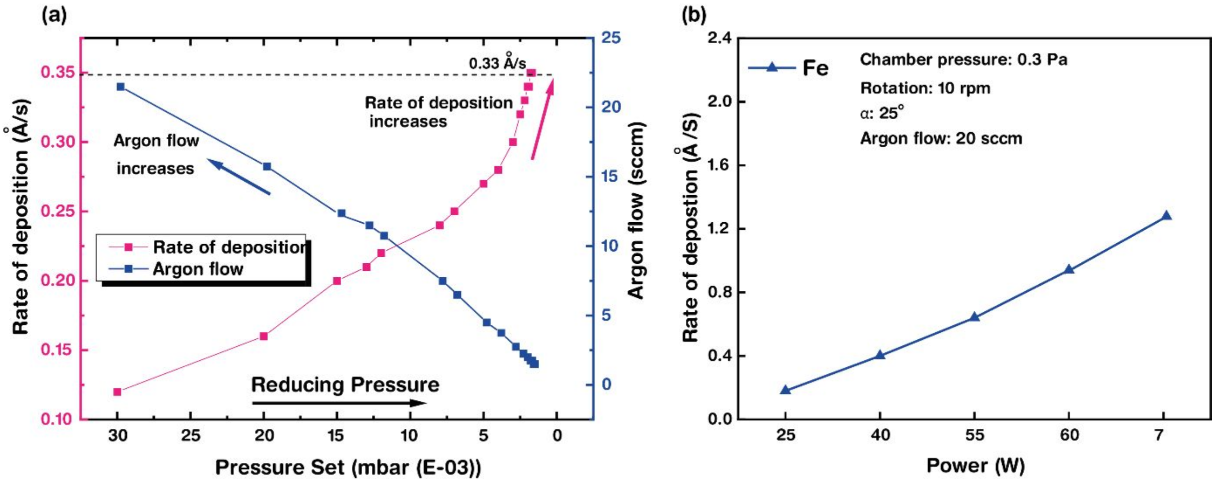

Figure S1: (a) Variation trend of rate of deposition of Fe target as a function of chamber pressure and argon gas inflow. (b) Variation trend of rate of deposition of Fe sputtering targets as a function of RF power

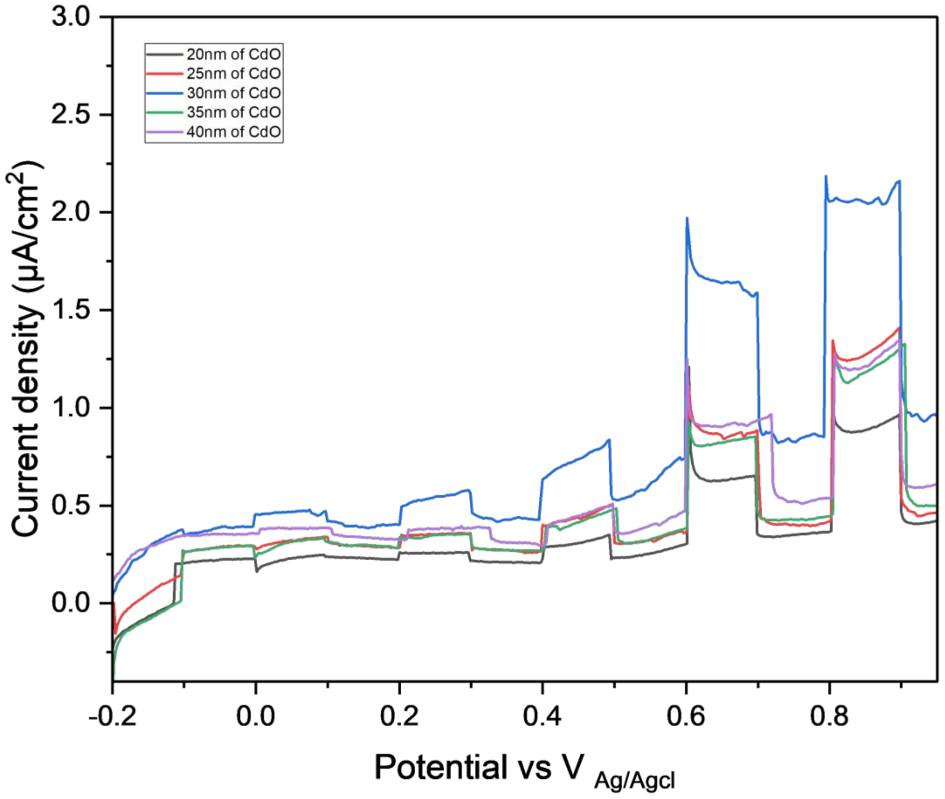

Figure S2: Linear sweep voltammetry of Cdo thin film (LSV of current density – potential vs Ag/AgCl) plot under 100 mW/cm^2 .
